# Defining quality indicators for practices, instruments, and programs across the JJ-TRIALS behavioral health services cascade

**DOI:** 10.1186/1940-0640-10-S1-A69

**Published:** 2015-02-20

**Authors:** Gail Wasserman, Gene Brody, Tisha Wiley

**Affiliations:** 1 Columbia University, New York, NY, 10027, USA; 2 Center for Family Research, University of Georgia, Athens, GA, 30602, USA; 3 Services Research Branch, National Institute on Drug Abuse, Rockville, MD, 20852, USA

## 

In order to address JJ-TRIALS goals of: a) improving behavioral health services for youth with substance use problems; and b) advancing the investigation of implementation efforts in the field of behavioral health, the JJ-TRIALS Workgroup on Evidence-Based Practices (EPA) was first charged with defining quality indicators for practices and programs. We limited that effort to programs, practices, and instruments relevant to the steps in the Behavioral Services Cascade (Screening, Referral and Linkage, Assessment, Prevention and Psychosocial Treatment) for five identified clinical problem areas (Substance use, Mood disorder, ADHD, Trauma exposure, HIV risk). Problem areas were selected as those of moderate prevalence among adolescents in community justice systems with problem substance use. Quality indicators were consistent with the AACAP Practice Parameters Clinical Standard, as reflecting either “rigorous empirical evidence” or “overwhelming clinical consensus” (American Academy of Child and Adolescent Psychiatry, 2013). In a series of directed literature reviews, we catalogued evidence-based programs and instruments addressing these problem areas that had been identified as most strongly supported by existing systematic reviews (e.g., SAMHSA, 2011) and then categorized them into tiers, based on their applicability for JJ-TRIALS efforts (e.g., number of TRIALS problem areas addressed, administration format, delivery setting, inclusion of family collaterals). These reviews identified 18 psychometrically sound screening instruments (3 tiers), 16 sound assessment instruments (4 tiers), 43 EB prevention programs (3 tiers), and 39 EB treatment programs (3 tiers). While the evidence base regarding programs that focus on cross-system linkage (e.g., from screening in a probation setting, with a subsequent referral to a behavioral health provider) is less established, EPA was able to designate three tiers of such programs, defined both by their soundness and their applicability to juvenile justice community settings. As a second set of quality indicators, we considered core content components (that may cut across particular instruments or programs). For assessment, these included eight elements essential for clinical treatment planning for adolescents (e.g., family relationships, readiness for change: American Society of Addiction Medicine, 2013). For treatment programs, these included treatment modalities identified as effectively addressing one or another of the TRIALS problem areas (e.g., CBT; Chorpita, et al., 2011). A final quality indicator for assessment and treatment considered procedural elements (that relate to how an instrument or program is used by a service provider), such as manualization, staff training, and fidelity monitoring (e.g., Brannigan, 2004; Howell & Lipsey, 2012). EPA workgroup products will be incorporated into future JJ-TRIALS training efforts; they will be used to generate menus of high-quality instrument and program options to help juvenile justice partners and the behavioral health agencies with which they collaborate to set implementation goals for participation in JJ-TRIALS.

